# Chronic Fluoxetine Impairs the Effects of 5-HT_1A_ and 5-HT_2C_ Receptors Activation in the PAG and Amygdala on Antinociception Induced by Aversive Situation in Mice

**DOI:** 10.3389/fphar.2020.00260

**Published:** 2020-03-11

**Authors:** Daniela Baptista-de-Souza, Lígia Renata Rodrigues Tavares, Elke Mayumi Furuya-da-Cunha, Paulo Eduardo Carneiro de Oliveira, Lucas Canto-de-Souza, Ricardo Luiz Nunes-de-Souza, Azair Canto-de-Souza

**Affiliations:** ^1^Department of Psychology, Federal University of São Carlos-UFSCar, São Carlos, Brazil; ^2^Joint Graduate Program in Physiological Sciences, UFSCar/UNESP, São Carlos, Brazil; ^3^Institute of Neuroscience and Behavior, Ribeirão Preto, Brazil; ^4^Laboratory of Pharmacology, School of Pharmaceutical Sciences, Univ. Estadual Paulista – UNESP, Araraquara, Brazil; ^5^Graduate Program in Psychology UFSCar, São Carlos, Brazil

**Keywords:** fluoxetine, 5-HT_1A_ and 5HT_2C_ receptors, serotonin, amygdala, periaqueductal gray matter, antinociception, mice

## Abstract

Growing evidence suggests an important role of fluoxetine with serotonin 5-HT_1A_ and 5-HT_2C_ receptors in the modulation of emotion and nociception in brain areas such as the amygdala and periaqueductal gray (PAG). Acute fluoxetine impairs 5-HT_2C_ (but not 5-HT_1A_) receptor activation in the amygdaloid complex. Given that fluoxetine produces its clinical therapeutic effects only when given chronically, this study investigated the effects of chronic treatment with fluoxetine on the effects produced by 5-HT_1A_ or 5-HT_2C_ receptors activation in the amygdala or PAG on fear-induced antinociception. We recorded the effects of chronic fluoxetine on serotonin and its metabolite 5-hydroxyindoleacetic acid (5-HIAA) levels as well as serotonin turnover; 5-HT_1A_ and 5-HT_2C_ receptor protein levels in the amygdala and PAG. Also, we evaluated the effects of chronic fluoxetine combined with intra-amygdala or intra-PAG injection of MK-212 (a 5-HT_2C_ agonist; 0.63 nmol) or 8-OH-DPAT (a 5-HT_1A_ agonist; 10 nmol) on the antinociceptive response in mice confined in the open arm of the elevated plus-maze (EPM). Nociception was assessed with the writhing test induced by intraperitoneal injection of 0.6% acetic acid. Results showed that fluoxetine (20 mg/kg, s.c.) enhanced the open-arm induced antinociception (OAA) and reduced the number of writhes in mice confined in the enclosed arm, featuring an analgesic effect. In addition, fluoxetine increased the expression of 5-HT_2C_ receptors and 5-HT levels whereas reduced its turnover in the amygdala. While fluoxetine did not change 5-HT and 5-HIAA levels, and its turnover in the PAG, it up-regulated 5HT_1A_ and 5-HT_2C_ receptors in this midbrain area. Chronic fluoxetine (5.0 mg/Kg, an intrinsically inactive dose on nociception) antagonized the enhancement of OAA produced by intra-amygdala or intra-PAG injection of MK-212. Fluoxetine also impaired the attenuation of OAA induced by intra-amygdala injection of 8-OH-DPAT and totally prevented OAA in mice that received intra-PAG 8-OH-DPAT. These results suggest that (i) 5-HT may facilitate nociception and intensify OAA, acting at amygdala 5-HT_1A_ and 5-HT_2C_ receptors, respectively, and (ii) fluoxetine modulates the OAA through activation of 5-HT_2C_ receptors within the PAG. These findings indicate that chronic fluoxetine impairs the effects of 5-HT_1A_ and 5-HT_2C_ receptors activation in the amygdala and PAG on fear-induced antinociception in mice.

## Introduction

Serotonin (5-HT) is involved in the etiology of numerous disease states, including depression, anxiety, panic disorders and painful conditions (Richardson, 1990; Supornsilpchai et al., 2006; Artigas, 2013a; Zangrossi and Graeff, 2014). The 5-HT receptors belong to the G-protein-coupled receptor superfamily (except for the 5-HT3 receptor, which is a ligand-gated ion channel) with at least 14 distinct members (Hoyer et al., 2002), however, the role of 5-HT_1A_ and 5-HT_2__*C*_ receptor subtypes in the modulation of emotional and pain responses have been more extensively studied (Zanoveli et al., 2010; Strauss et al., 2013; Baptista-de-Souza et al., 2014; Furuya-da-Cunha et al., 2016).

Previous findings have been showing that although with antagonistic intracellular mechanisms, 5-HT_1A_ (inhibitory G-coupled protein receptor) and 5-HT_2C_ (stimulatory G-coupled protein receptor) (Shih et al., 1991; Azmitia, 2007; Pytliak et al., 2011) may interact mutually in the modulation of behavioral and endocrine responses (Hensler and Truett, 1998; Valdez et al., 2002).

Selective serotonin reuptake inhibitors (SSRIs; e.g., fluoxetine) interfere in the modulation of emotions (Argyropoulos et al., 2000; Nash and Hack, 2002; Krishnan and Nestler, 2008). A body of evidence has been demonstrating that SSRIs provoke the relief of painful symptoms in patients with several pain syndromes, which are frequently associated to emotional disorders, suggesting that these states share biochemical mechanisms (Blackburn-Munro and Blackburn-Munro, 2001; Suzuki et al., 2004; Kostov and Schug, 2018). SSRIs also attenuate defensive responses in animal models, provoking anxiolytic and panicolytic effects (Zangrossi and Graeff, 2014). In addition, they attenuate pain response in some animal tests (Schreiber and Pick, 2006) such as the tail-flick and writhing tests for rodents (Pedersen et al., 2005).

The influence of emotional states such as anxiety and fear on pain reaction has been widely investigated in animal models (Siegfried et al., 1990; Fields, 2004; Baptista et al., 2009; Furuya-da-Cunha et al., 2016) and the exposure to the elevated plus-maze (EPM), a widely used animal model of anxiety (Lister, 1987; Carobrez and Bertoglio, 2005) has been employed to investigate the underlying mechanisms of the anxiety/fear-induced antinociception phenomenon (Lee and Rodgers, 1990). In this context, previous studies have been showing the role of serotonin at 5-HT_1A_ and 5-HT_2C_ receptors in the modulation of the antinociception induced by the EPM open-arm confinement (OAA) in mice (Nunes-de-Souza et al., 2000; Baptista-de-Souza et al., 2018; Tavares et al., 2018).

Regarding the neurobiological substrate of the anxiety/fear-induced antinociception, several studies have pointed out the crucial role of the amygdaloid complex and the midbrain periaqueductal gray (Fields, 2000; Neugebauer, 2015). These areas are markedly involved in the modulation of some types of environmentally induced antinociception, such as the OAA (Canto-de-Souza et al., 1998; Mendes-Gomes et al., 2011; Biagioni et al., 2012; Coimbra et al., 2017). In this context, many studies have demonstrated that the analgesia induced by stimulation of the PAG is related to areas with a high concentration of serotonergic receptors (Lohof et al., 1987; Coimbra and Brandao, 1997; Brandao et al., 1999; Zhang et al., 2000; Lu et al., 2010). In this sense, we have previously reported the role of 5-HT_1A_ and 5-HT_2C_ receptors in reverse and accentuate, respectively, the fear-induced antinociception in mice (Baptista et al., 2012; Furuya-da-Cunha et al., 2016; Tavares et al., 2018).

Interestingly, 5-HT_1A_ and 5-HT_2C_ receptor subtypes seem to play antagonistic intracellular mechanisms (Shih et al., 1991; Azmitia, 2007; Pytliak et al., 2011). For instance, it has been demonstrated that activation of these two serotonin receptors provokes opposite effects on behavioral and endocrine responses (Hensler and Truett, 1998; Zhang et al., 2001; Valdez et al., 2002). Furthermore, electrophysiology studies have demonstrated that acute and chronic treatment with fluoxetine can differentially modulate the activity of 5HT_1A_ (Subhash et al., 2000; Descarries and Riad, 2012) and 5-HT_2C_ receptors (Dremencov et al., 2006; Mongeau et al., 2010). Moreover, the influence of this interaction on emotional responses was demonstrated by several studies, wherein the anxiolytic and panicolytic responses induced by intra-PAG infusions of 5-HT_1A_ and 5-HT_2_ agonists receptors were accentuated after chronic administration of fluoxetine (de Bortoli et al., 2006; Zanoveli et al., 2007, 2010).

Regarding the amygdala, studies have shown that the activation of the 5-HT_1A_ receptors of the basolateral amygdala nucleus (BLA) produces anxiolytic effects in rats exposed to the elevated T-maze (ETM) (Strauss et al., 2013; Vicente and Zangrossi, 2014). On the other hand, Vicente and Zangrossi (2014) also demonstrated that chronic systemic treatment with imipramine and fluoxetine causes desensitization of 5-HT_2C_ receptors in the BLA of rats exposed to the ETM. In this context, Tavares et al. (2018) have recently shown that acute systemic fluoxetine blocked the OAA enhancement induced by intra-amygdala microinjection of MK-212 (a 5-HT_2C_ receptor agonist), suggesting that this SSRI may be acting as a 5-HT_2C_ receptor antagonist. In favor of that interpretation, Tavares et al. (2018) also showed that intra-amygdala combined injection of SB 242084, a selective 5-HT_2C_ receptor antagonist, prior to local injection of MK-212 produced a similar effect on OAA.

Even though these findings above have described specificities concerning fluoxetine mechanisms, it remains unknown the mechanisms by which SSRIs induce rapid neurochemical changes, but take a long time (e.g., weeks) to produce therapeutic efficacy in mood or anxiety disorders. Several studies have shown that this temporal dynamic depends on neuroplastic changes induced by chronic administration of fluoxetine (Klomp et al., 2015; Rafa-Zablocka et al., 2018; Ampuero et al., 2019). In this sense, serotonergic receptors would be considered as agents to perform these changes. For this reason, a better understanding of how fluoxetine and 5-HT_1A_ and 5-HT_2C_ receptors interplay may be important to the best usage of this class of drugs on anxiety, depression and pain disorders. Therefore, we hypothesized that chronic treatment with fluoxetine alters the role of 5-HT_1A_ and 5-HT_2C_ receptors located in the amygdaloid complex and PAG in the modulation of OAA in mice.

Here, we evaluated the effects of chronic fluoxetine on serotonin, 5-HIAA levels, the serotonin turnover, 5-HT_1A_, and 5HT_2__*C*_ protein levels within the amygdala and the PAG; and the effects of chronic systemic fluoxetine combined with acute intra-amygdala or intra-PAG injections of MK-212 (a 5-HT_2C_ receptor agonist), and the 8-OH-DPAT (a 5-HT_1A_ receptor agonist) on OAA in mice.

## Materials and Methods

### Animals

Four hundred thirty-three adult male Swiss mice (Federal University of São Carlos, SP, Brazil), weighing 25–30 g, were housed in groups of 10 per cage (cage size: 41 cm × 34 cm × 16 cm). They were maintained under a normal 12:12 h light-dark (LD) cycle (lights on 7:00 a.m.) in a temperature (24 ± 1 °C) controlled environment. Food and drinking water were freely available except during the brief test periods. The experiments were carried out during the light phase of the LD cycle (9:00 am – 4:00 pm). Different batches of experimentally naïve mice were used for each experiment.

### Ethics Statement

The experiments reported in this study were performed in compliance with the recommendations of the Brazilian Guidelines for Care and Use of Animals for Scientific and Educational Purposes, elaborated by The National Council of Control of Animal Testing (CONCEA). This study was also approved by the Ethics Committee on the Use of Animals of the Federal University of São Carlos (Res. 046/2009; 021/2014). All endeavors were made to reduce animal suffering and to minimize the number of animals used.

### Drugs

Fluoxetine hydrochloride, Sigma, (5.0, 10, or 20 mg/Kg, s.c.) were prepared in physiological saline (0.9% NaCl) (Singh et al., 2001). 8-OH-DPAT [(±)-8-hydroxy-2-(di-n-propylamino) tetralin hydrobromide; Sigma, (10 nmol)] (5-HT_1A_ receptor agonist); MK-212 [6-chloro-2-(1-piperazinyl)] pyrazine hydrochloride, Tocris Cookson Inc. (0.63 nmol) (5-HT_2C_ receptor agonist). Doses used for the intra-amygdala and intra-PAG treatments were based on previous studies (Nunes-de-Souza et al., 2000; Gomes and Nunes-De-Souza, 2009; Vicente and Zangrossi, 2012; Baptista-de-Souza et al., 2018; Tavares et al., 2018). The doses were prepared in saline or vehicle (physiological saline with 2 % of Tween 80) and a final volume of 0.1 μl was injected.

### Surgery and Microinjection

For the amygdaloid complex, two stainless-steel guide cannulas (25-gauge × 7 mm; Insight Instruments, Brazil) were implanted under ketamine + xylazine anesthesia (100 mg/kg and 10 mg/kg, i.p.) in a stereotaxic frame (Insight Instruments, Brazil). The guide cannulas were fixed to the skull with dental acrylic. The bregma was considered the reference point, and the following coordinates were used to locate the target site: anterior/posterior, -1.3 mm; medial/lateral, ±3.3 mm; dorsal/ventral, -2.8 mm (Paxinos and Franklin, 2001). Stereotaxic coordinates for the target site in the PAG were 4.1 mm posterior to bregma, +1.3 mm lateral to the midline and 1.2 mm ventral to the skull surface. One guide cannula for the PAG was implanted at an angle of 26° to the vertical and was aimed to terminate 2 mm from the target site. To reduce the incidence of occlusion, a dummy cannula (33-gauge stainless steel wire; Fishtex^®^, Brazil) was inserted into the guide cannula at the time of surgery. During the surgery, animals received ketoprofen (benzeneacetic acid, 5 mg/kg, i.p.) and ceftriaxone (ceftriaxone sodium *hemieptahydrate*, 4 mg/kg, i.p.) (Garber, 2011; Stepanovic-Petrovic et al., 2014). Before behavioral tests, mice were allowed 4–5 days to recover from surgery. The microinjection procedure was performed on the 21st day of treatment with fluoxetine, 30 min after the last injection. Solutions were injected into the amygdala and the PAG by a microinjection unit (33-gauge stainless steel cannula, Insight Instruments, Brazil), that extended 2 mm beyond the tip of the guide cannula. The microinjection unit was connected to a 10 μl Hamilton microsyringe via polyethylene tubing (PE-10) and the rate of flow was controlled by an infusion pump (BI 2000–Insight Instruments, Brazil) programmed to deliver 0.1 μl of each solution over a period of 60 s. The microinjection procedure consisted of gently restraining the mouse, inserting the injection unit, infusing the solution for 60 s and keeping the injection unit in place for 90 s. The movement of a small air bubble in the PE-10 tubing, during and after the microinjection, confirmed the delivery of the solution (Nunes-de-Souza et al., 2000).

### Apparatus and General Procedure

The basic EPM design was very similar to that originally described by Lister (1987). It comprised two open arms (OA: 30 cm × 5 cm × 0.25 cm) and two enclosed arms (EA: 30 cm × 5 cm × 15 cm) that extended in a cross from a common central platform (5 cm × 5 cm), raised to a height 38.5 cm above floor level. Confinement to an OA or EA was achieved by placing an easily removable gate at the proximal end of each arm of the EPM. All testing was conducted under moderate illumination (77 lux; measured on the central platform of the EPM) during the light phase of the LD cycle.

Nociception was assessed by the writhing test as previously described (Vander Wende and Margolin, 1956). In the present study, writhes were induced by injecting 0.1 mL/10 g body weight (b.w.) of 0.6% acetic acid i.p, 5 min after the intra-amygdala and intra-PAG drug injection. They were then individually confined to either an OA or an EA of the EPM for 5 min, during which the number of writhes was recorded. Between subjects, the maze was thoroughly cleaned with 20% ethanol and dried with a cloth. All sessions were video recorded with a camera linked to a monitor in an adjacent laboratory. This experimental protocol was repeated in all experiments described below.

### Experimental Procedures

#### Experiment 1. Effects of Chronic Fluoxetine Treatment on OAA in Mice

This protocol aimed to investigate the effects of chronic fluoxetine treatment on antinociception induced by the confinement of mice to the open arms of the EPM, to obtain a dose-response curve of fluoxetine treatment. Sixty-two mice received daily subcutaneous injection of saline or fluoxetine (5, 10 or 20 mg/Kg) for 21 days. Thirty minutes after the last s.c. injection, each animal received an i.p. injection of 0.6% acetic acid (0.1 ml/10 g b.w.), as a nociceptive stimulus, and, 5 min later, was confined either in the enclosed (EA) (*n* = 31) or open arm (OA) (*n* = 31) of the EPM for 5 min to record the number of writhes.

#### Experiment 2. Effects of Chronic Fluoxetine Treatment on Serotonin and 5-HIAA Levels and 5-HT Turnover Within the Amygdala and PAG

This protocol aimed to investigate whether chronic fluoxetine treatment would increase 5-HIAA levels and 5-HT turnover within the amygdala and PAG. Fifty-three mice received daily subcutaneous injection of saline (*n* = 6) or fluoxetine (5, 10, or 20 mg/Kg; *n* = 6–7) for 21 days. On the last day of treatment and 30 min after the last injection, they were confined either in the enclosed (EA) or open arm (OA) of the EPM and were decapitated in a guillotine and their brains were extracted and stored.

#### Experiment 3. Effects of Chronic Fluoxetine Treatment on 5-HT_1A_ and 5-HT_2C_ Protein Levels Within the Amygdala and PAG

This protocol aimed to investigate whether chronic fluoxetine treatment changes 5-HT_1A_ and 5-HT_2C_ protein levels within the amygdala and PAG. Twenty-four mice received daily subcutaneous injection of saline (*n* = 8) or fluoxetine (5 or 20 mg/Kg) (*n* = 16) for 21 days, and, 30 min after the last injection, the animals were decapitated in a guillotine and their brains were extracted and stored.

##### Experiment 4. Effects of Combined Treatment With Chronic Systemic Fluoxetine Injection and Acute Bilateral Intra-Amygdala Microinjection of MK-212 or 8-OH-DPAT on OAA

**FIGURE 1:**
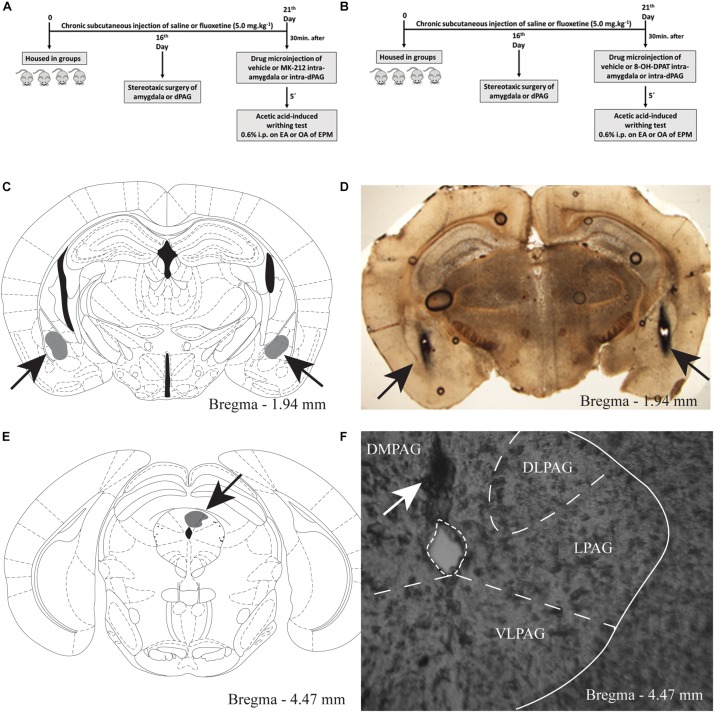
**A** and **B** Timeline of the protocol showing the treatment with chronic systemic fluoxetine followed by intra-amygdala or intra-PAG drug injection in mice exposed to the enclosed or open arms of the EPM, **(C)** target sites for microinjection into the amygdala. Schematic representation of microinjection sites into the amygdala. Gray region represent the sites of drug infusion that were on-target in the amygdala **(D)** photomicrograph of a coronal section of a representative subject showing an injection site in the mouse amygdala. **E** The gray region represent the sites of drug infusion that were on-target in the periaqueductal gray. Photomicrography of a coronal section of a representative subject showing an injection site in the mouse PAG **(F).**

This protocol aimed to investigate the effects of the interaction of chronic fluoxetine and the activation of 5-HT_2C_ or 5-HT_1A_ receptors within the amygdala on OAA. One hundred and sixty-four mice received daily subcutaneous injection of saline (*n* = 82) or fluoxetine (5 mg/Kg; *n* = 82) for 21 days. On the 16th day of the chronic treatment, all mice underwent stereotaxic surgery for implantation of guide cannulas in the amygdala. On the 21st day, 30 min after the last s.c. injection of saline or fluoxetine, animals received bilateral intra-amygdala microinjection of vehicle (*n* = 81), MK-212 (0.63 nmol/0.1 μL; *n* = 42) or 8-OH-DPAT (10 nmol/0.1 μL; *n* = 38) and, 5 min later, each mouse received 0.6% acetic acid i.p. injection (0.1 mL/10 g b.w.). Five minutes after acetic acid injection, each mouse was confined either in the OA or in EA of the EPM to record the number of writhes (for details, see Figure 1A).

##### Experiment 5. Effects of Combined Treatment With Chronic Fluoxetine Injection and Intra-PAG Microinjection of MK-212 or 8-OH-DPAT on OAA

This protocol aimed to investigate the effects of the interaction of chronic fluoxetine and the activation of 5-HT_2C_ or 5-HT_1A_ receptors within the PAG on OAA. One hundred and thirty-three mice received subcutaneous injection of saline (*n* = 66) or fluoxetine (5 mg/Kg; *n* = 67) for 21 days. On the 21st day, 30 min after the last s.c. injection of saline or fluoxetine, animals received intra-PAG injection of vehicle (*n* = 67), MK-212 (0.63 nmol/0.1 μL; *n* = 34) or 8-OH-DPAT (10 nmol; *n* = 32) and, 5 min later, each mouse received 0.6% acetic acid i.p. injection (0.1 mL/10 g b.w.). Five minutes after acetic acid injection, animals were individually confined either in the EA or OA of the EPM to record the number of writhes (for details, see Figure 1A).

### Histology

At the end of testing, the animals of experiments 4, 5 received a 0.1 μl infusion of 1% methylene blue into the amygdala or PAG, exactly as described above for drug microinjection. The animals were then euthanized by anesthetic overdose (300 mg/kg ketamine + 30 mg/kg xylazine, i.p.), their brains removed, and injection sites were verified histologically according to the atlas of Paxinos and Franklin (2001). Data from animals with injection sites outside the amygdaloid complex and PAG were excluded from the study.

### Tissue Processing

For experiments 2 and 3, the brains were extracted and frozen in isopentane and stored at - 80 °C until dissection. One-millimeter coronal slices of the brain (rostral face beginning approximately 2.0 mm from Bregma, based on the atlas of Paxinos and Franklin (2001) were cut in a cryostat at -20°C. Tissue punches (using a blunt 15-gauge needle) were obtained from the amygdala and periaqueductal gray (PAG).

### High-Performance Liquid Chromatography (HPLC) Assay

For determination of serotonin (5-HT) levels and its metabolite 5-hydroxyindole acetic acid (5-HIAA), the samples were homogenized in 0.1 M perchloric acid (60 μl), centrifuged at 1300 rpm for 20 min at 4°C; 30 μl of filtrated supernatant was injected into the chromatography system for quantification of the neurotransmitter and its metabolite by electrochemical detection in high-performance liquid chromatography (HPLC). The chromatograph (Waters and Alliance) consists of a Symmetry C18, 5 μm (250 mm × 4.6 mm) and 100 Å pore-diameter particle size, coupled with glassy-carbon electrochemical detector. The potential difference was set at 800 mV versus an Ag/AgCl reference electrode. The mobile phase, in a flow of 0.8 mL/min, with a citric acid constitution (0.05 M), heptane sulfonic acid (1.2 mM), pH 3.2, and after was added methanol (17%) for adjusting for pH.

The calibration curve was constructed with standard internal solutions of 1, 2.5, 5, 10, 25, 50, 100, 200, 400, and 600 ng/mL which were injected into the chromatograph in triplicate. The limit of detection and quantitation, as already standardized in this apparatus were: 1.0 to 3.5 for serotonin and 1.3 and 4.26 ng/mL for 5-HIAA. Finally, the levels of substances were corrected according to the mass from the dissected samples and were expressed as ng of substance per milligram of tissue. As an activation parameter, we used the serotonin turnover rate. The turnover was calculated by the ratio between metabolite and its respective neurotransmitter (Chi et al., 1999).

### Western Blot

After the area dissection, samples were sonicated in 1% sodium dodecylsulfate (SDS), and the protein concentration was determined by Bio-Rad DC protein assay (Bio-Rad Laboratories Inc., Hercules, CA, United States). Twenty micrograms of protein of each sample were boiled for 5 min in a loading buffer containing 5% of b-Mercaptoethanol and 0.002% of Bromophenol Blue and subjected to electrophoresis in 9% SDS-polyacrylamide minigels for 50 or 60 min at 200 V using the BioRad Mini-PROTEAN tetra cell with Bio-Rad Powerpac basic supply (Bio-Rad Laboratories Inc.). Proteins were electrophoretically transferred to low-fluorescence polyvinylidene fluoride membranes (LFPVDF) using the *Trans-*Blot Turbo system (Bio-Rad Laboratories Inc.). Membranes were washed in TBS-Tween 20 (TBS-T) buffer, pH 7.4 and blocked with 5% non-fat dry milk TBS-T, pH 7.4, for 1 h at room temperature. Membranes were then incubated with antibodies for both 5HT_1A_ (1:1000 Sigma-Aldrich, Cat. n°SAB4501469; polyclonal anti-rabbit, synthesized peptide derived from human, mol. wt 45 kDa) or anti-5-HT_2C_ (1:1000 Abcam, Cat. n°AB137529; polyclonal anti-rabbit, corresponding to a region within amino acids 387–436 of Human 5HT_2C_ Receptor, mol. wt 50 kDa) overnight at 4°C. Next, membranes were washed extensively with TBS-T solution, incubated for 60 min with HRP-conjugated anti-rabbit IgG (1:2000; Amersham Pharmacia Biotech^®^), and washed extensively again in TBS-T. Protein bands were visualized on a Kodak Biomax Light film with enhanced chemiluminescence (ECL plus, Amersham Pharmacia Biotech^®^). Equal protein loading was confirmed by stripping the blots and reprobing them with a monoclonal β-actin antibody (1:500 Santa Cruz Biotechnology^®^), followed by incubation with secondary antibody and visualization as described above. The films were scanned in transparency mode and the volume of the bands was quantified using Image-Master^®^ software (Amersham Pharmacia Biotech^®^) with the subtraction of background. As determined by preliminary experiments, all assays were conducted under conditions in which densitometric signal intensity was linear with protein concentration.

### Statistical Analysis

The data of experiments 1 and 2 were analyzed by two-way analysis of variance (ANOVA) (treatment × type of confinement) and experiment 3 was analyzed by one-way ANOVA (treatment factor). Data of experiments 4 and 5 were analyzed by three-way ANOVA (systemic treatment × intra-PAG or intra-amygdala treatment × type of confinement). In cases of significant interactions, single effects of the factors were analyzed by one-way ANOVAs followed by the Duncan test. A *p*-value of 0.05 or less was required for significance.

## Results

For experiments 4 and 5, only mice with microinjection sites located bilaterally in the amygdala and unilaterally in the PAG were included in the study. Figures 1C, 1E show a schematic representation of histological results according to the Mouse Brain Atlas (2001). Gray areas represent on-target of infusion within the amygdala and PAG. Figures 1D,F show photomicrographs of a midbrain coronal section of a representative subjects showing an injection site within the amygdala and PAG, the arrows represents the injection sites.

**FIGURE 2:**
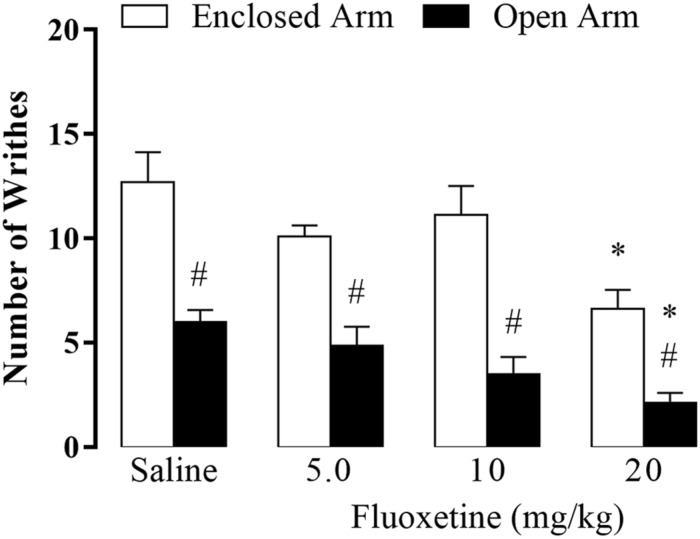
Effect of chronic treatment of fluoxetine (5, 10, and 20 mg/kg, s.c.) on nociceptive response of EA- and OA-confined mice (*n* = 7–9). Data are presented as mean + SEM. ^#^*p* < 0.05 vs. EA-confined group. *p < 0.05 vs. respective control group.

### Experiment 1: Fluoxetine Enhanced OAA

Two-way ANOVA revealed significant effects for the type of confinement factor [*F*(1,54) = 83.10, *p* < 0.05], for the treatment factor (*F*(3,54) = 9.57, *p* < 0.05], and no interaction between factors type of confinement and treatment factor [*F*(3,54) = 1.16, *p* = 0.33]. Duncan’s *post hoc* test indicated that the number of writhes was significantly higher in EA-confined animals than in the OA group. Furthermore, animals that received fluoxetine (20 mg/kg) and were confined in the EA or OA displayed a lower number of writhes compared to their respective control groups (Figure 2).

### Experiment 2: Effects of Chronic Fluoxetine Treatment on Serotonin, 5-HIAA Levels and Turnover Within the Amygdala and PAG in Mice

#### Amygdala

**FIGURE 3:**
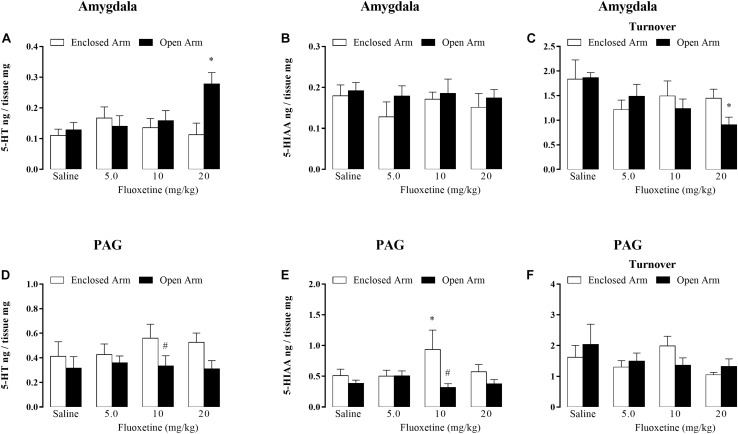
Effects of chronic treatment with fluoxetine (5, 10, and 20 mg/kg, s.c.) on the levels of 5-HT **(A)**, 5-HIAA (**B),** and serotonin turnover **(C)** within the amygdala. Levels of serotonin **(D)**, 5-HIAA (**E),** and serotonin turnover **(F)** in nanograms *per* tissue within the PAG in mice confined in the open or enclosed arms of the EPM (*n* = 5–7). Data are represented as mean + SEM. ^#^*p* < 0.05 vs. EA-confined group, ^∗^*p* < 0.05 vs. respective control group.

5-HT levels: two-way ANOVA revealed significant effects for confinement versus treatment interaction [*F*(3,44) = 3.55, *p* < 0.05], but not for treatment factor [*F*(3,44) = 2.06, *p* = 0.17] and type of confinement factor [*F*(1,44) = 3.90, *p* = 0.08] (Figure 3A).

5-HIAA levels: two-way ANOVA revealed no significant effects for type of confinement factor [*F*(1,44) = 1.60, *p* = 0.21]; treatment [*F*(3,44) = 0.55, *p* = 0.64] for confinement versus treatment interaction [*F*(3,44) = 0.20, *p* = 0.89] (Figure 3B).

5-HT turnover: two-way ANOVA showed significant effects for treatment factor [*F*(4,44) = 3.22, *P* < 0.05], but not for type of confinement factor [*F*(1,44) = 0.54, *p* = 0.89] and treatment versus type of confinement interaction [*F*(3,44) = 1.14, *p* = 0.34] (Figure 3C).

*Post hoc* analyses confirmed that the serotonin levels were significantly higher in mice that received 20 mg/kg of fluoxetine and were confined in OA than in EA-confined animals (Figure 3A). Duncan’s *post hoc* test also revealed a significantly lower serotonin turnover in mice that received 20 mg/kg of fluoxetine (Figure 3C).

#### PAG

5-HT levels: two-way ANOVA revealed significant effects for the type of confinement factor [*F*(1,48) = 5.55, *p* < 0.05) and did not for treatment [*F*(3,45) = 0.30, *p* = 0.82] and interaction [*F*(3,45) = 0.41, *p* = 0.74] (Figure 3D).

5-HIAA levels: two-way ANOVA revealed effects for the type of confinement factor [*F*(1,45) = 6.84, *p* < 0.05] and did not for treatment [*F*(3,46) = 0.57, *p* = 0.63] and interaction [*F*(3,46) = 2.45, *p* = 0.07] (Figure 3E).

Duncan’s *post hoc* test confirmed that the 5-HIAA levels were significantly lower in the mice that received 10 mg/kg de fluoxetine and were confined in OA than EA-confined animals (Figure 3E).

5-HT turnover: two-way ANOVA revealed no significant effects for type of confinement factor [*F*(1,44) = 0.09, *p* = 0.75]; treatment [*F*(3,44) = 1.46, *p* = 0.23] for confinement versus treatment interaction [*F*(3,44) = 0.86, *p* = 0.46].

### Experiment 3: Effects of Chronic Systemic Fluoxetine on 5-HT_1A_ and 5-HT_2C_ Protein Levels in the Amygdala and PAG

#### Amygdala

**FIGURE 4:**
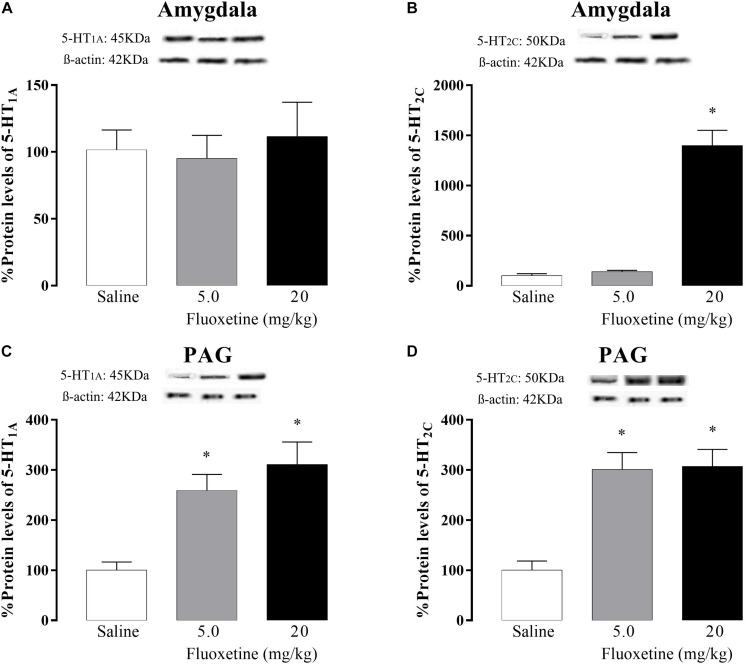
Effects of chronic treatment (21 days) with fluoxetine (5 and 20 mg/kg) on the 5-HT_1A_ and 5-HT_2C_ protein levels receptors in the amygdala (**A** and **B**, respectively) and PAG (**C** and **D**, respectively). ^∗^*p* < 0.05 vs. respective control group.

5-HT_1A_ receptor: One-way ANOVA revealed no significant differences [F(2,24) = 0.30; *p* = 0.74] (Figure 4A).

5-HT_2C_ receptor: One-way ANOVA revealed a significant difference [F(2,23) = 77.18; *p* < 0.05] and *post hoc* test confirmed that animals that received the 20 mg/kg of fluoxetine showed an increase in 5-HT_2__*C*_ receptor protein levels compared to the control group (Figure 4B).

### PAG

5-HT_1A_ receptor: one-way ANOVA revealed significant differences [F (2,18) = 12.49, *p* < 0.05] and *post hoc* test confirmed that both doses of fluoxetine increased 5-HT_1A_ receptor protein levels compared to the control group (Figure 4C).

5-HT_2C_ receptor: one-way ANOVA revealed significant difference [*F*(2,23) = 16.40, *p* < 0.05] and *post hoc* analyses confirmed that both doses of fluoxetine increased 5-HT_2C_ receptors protein levels compared to the control group (Figure 4D).

### Experiment 4: Chronic Systemic Fluoxetine Impairs the Effects of Intra-Amygdala Microinjection MK-212 or 8-OH-DPAT on OAA

#### Chronic Fluoxetine × Intra-Amygdala MK-212

Three-way ANOVA revealed statistically significant main effects for type of confinement factor [*F*(1, 69) = 57.90, *p* < 0.05] and for confinement × chronic systemic treatment factor × intra-amygdala treatment interactions [*F(*2,69) = 5.62, *p* < 0.05], but not for chronic treatment [*F*(1,69) = 0.19, *p* = 0.65], intra-amygdala treatment [*F*(1,69) = 2.38, *p* = 0.13], interaction between confinement and chronic treatment [*F*(1,69) = 2.67, *p* = 0.10], confinement and intra-amygdala treatment [*F*(1,69) = 0.39 *p* = 0.53], and between treatments [*F*(1,69) = 0.64, *p* = 0.42].

**FIGURE 5:**
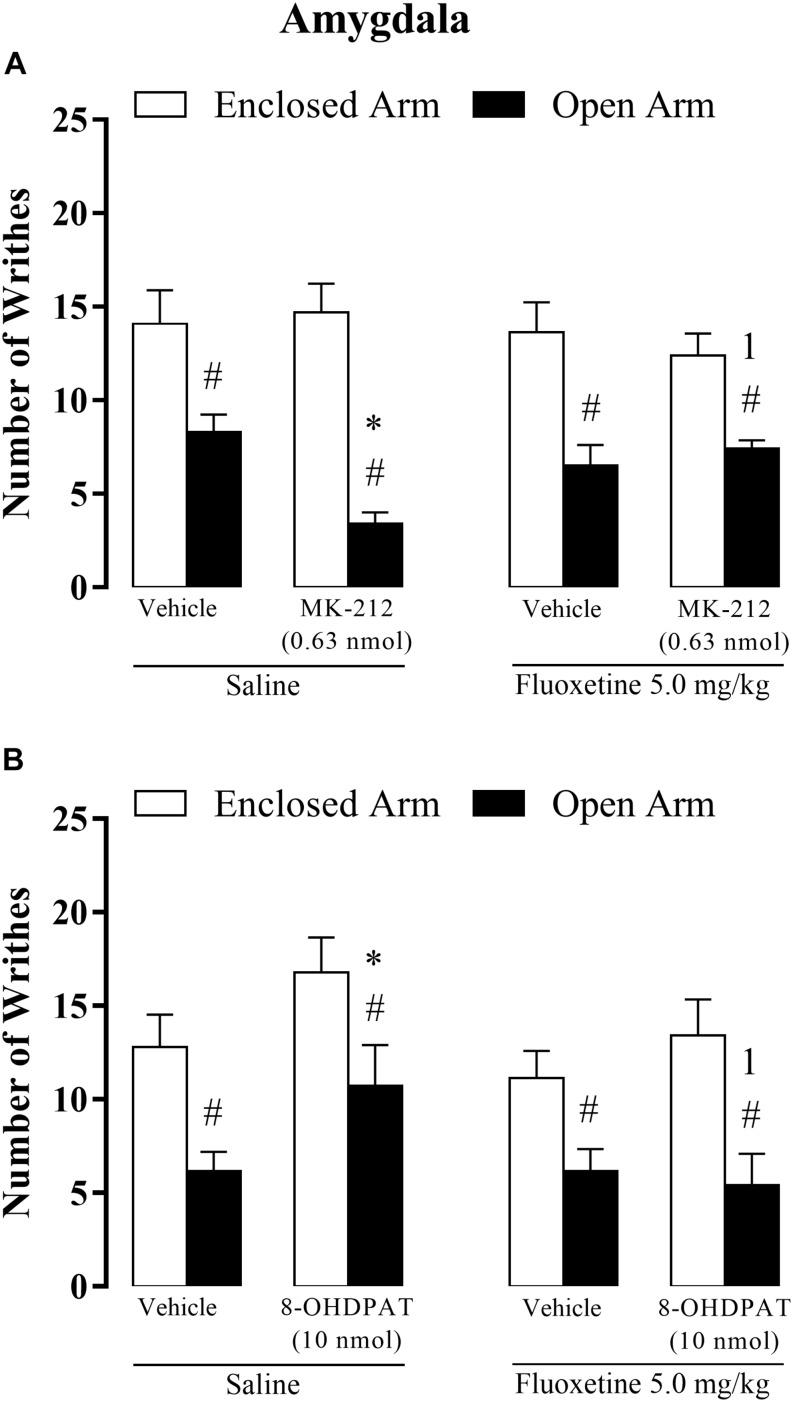
Effects of the combined injections of chronic systemic fluoxetine (5.0 mg/kg) followed by bilateral intra-amygdala microinjections of 8-OH-DPAT (10 nmol/0.1 μL) **(A)** and MK-212 (0.63 nmol/0.1 μL) **(B)** on OAA in mice. All data are presented as mean + SEM (*n* = 7–12). ^#^*p* < 0.05 compared to EA-confined group. ^∗^*p* < 0.05 compared to the respective vehicle group. ^1^*p* < 0.05 compared to the saline + 8-OH-DPAT group or saline + MK-212 group. Three-way ANOVA followed by Duncan’s *post hoc* test.

Again, the *post hoc* test confirmed that OA-confined animals displayed a lower number of writhes than in the EA-confined group. In addition, intra-amygdala injection of MK-212 increased OAA, but only in animals that received saline systemically. In other words, chronic fluoxetine prevented the enhancement of OAA-induced by intra-amygdala injection of MK-212 (Figure 5A).

#### Chronic Fluoxetine × Intra-Amygdala 8-OH-DPAT

Three-way ANOVA revealed significant effects for the type of confinement factor [*F*(1,72) = 30.97, *p* < 0.05], systemic treatment factor [*F*(1, 72) = 4.50, *p* < 0.05], and for intra-amygdala treatment factor [*F*(1, 72) = 4.96, *P* < 0.05].

Although, the statistical analysis did not reveal significance for interaction between confinement and chronic treatment [*F*(1,72) = 0.008, *p* = 0.97], confinement and intra-amygdala treatment [*F*(1,72) = 0.20 *p* = 0.65], treatments systemic and intra-amygdala [*F*(1,72) = 2.50, *p* = 0.11], and for interaction local of confinement, treatment systemic and intra-amygdala [*F(*1,72) = 0.74, *p* = 0.39].

Duncan’s *post hoc* test confirmed the lower number of writhes in the OA-confined group than in the EA-confined group. Also, for OA-confined animals, the saline + 8-OH-DPAT group increased the number of writhes compared with saline + vehicle-treated animals. Importantly, fluoxetine did not produce any effect *per se* on nociceptive response either in OA- or EA-confined animals. Overall, these data indicate that the attenuation of the OAA, observed in mice treated with saline + 8-OH-DPAT, was prevented by chronic fluoxetine treatment (Figure 5B).

### Experiment 5: Chronic Fluoxetine Treatment Alters the Effects of Intra-PAG Microinjection of MK-212 or 8-OH-DPAT and on OAA in Mice

#### Chronic Fluoxetine × Intra-PAG MK-212

Three-way ANOVA revealed significant effects for type of confinement factor [*F*(1,85) = 100.21, *p* < 0.05], intra-PAG treatment [*F*(2,85) = 3.13, *p* < 0.05] and systemic × intra-PAG × type of confinement interaction [*F*(2,85) = 3.72, *p* < 0.05], but not for systemic treatment [*F*(1,85) = 0.65 *p* = 0.41], confinement and chronic treatment [*F*(1,85) = 1.27, *p* = 0.26], confinement and intra-PAG treatment [*F*(2,85) = 2.52, *p* = 0.08], treatments systemic and intra-PAG [*F*(1,85) = 0.30, *p* = 0.73].

**FIGURE 6:**
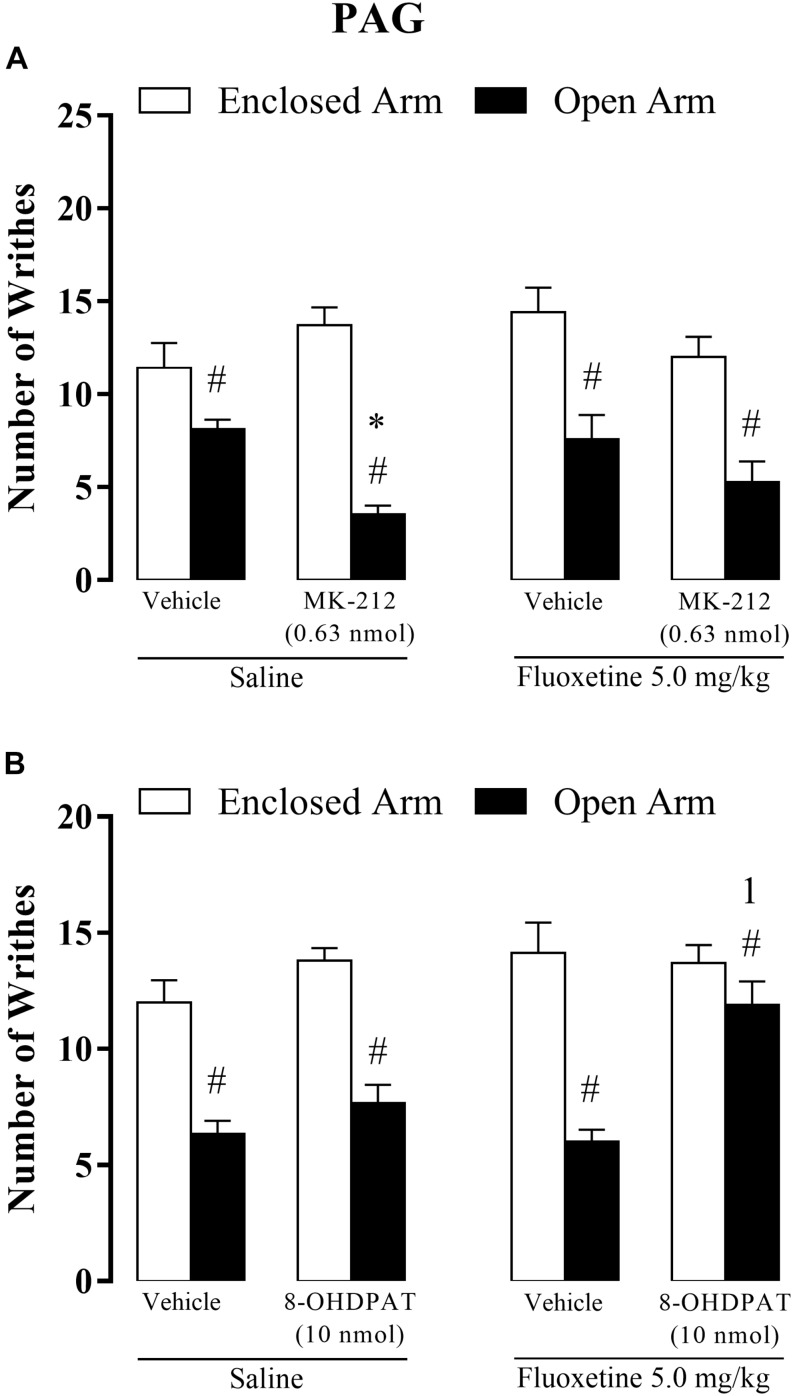
Effects of combined injections of systemic fluoxetine (5 mg/kg) and intra-PAG 8-OH-DPAT (10 nmol/0.1 μl) **(A)** or MK-212 (0.63 nmol/0.1 μL) **(B)** on OAA in mice (*n* = 7–10). Data are presented as mean + SEM. ^#^*p* < 0.05 vs. EA-confined group. ^∗^*p* < 0.05 vs. respective control group. ^1^*p* < 0.05 vs. saline + MK-212 group or saline + 8-OH-DPAT.

*Post hoc* analyses confirmed that the number of writhes was significantly higher in EA-confined animals than in the OA group and that OA-confined animals treated with MK-212 (saline + MK-212) exhibited a lower number of abdominal contortions than the control group (saline + vehicle). However, the enhancement of OAA induced by intra-PAG MK-212 was prevented by chronic fluoxetine. Importantly, fluoxetine *per se* (fluoxetine + vehicle) did not significantly change nociceptive response in EA- or OA-confined animals (Figure 6A).

#### Chronic Fluoxetine × Intra-PAG 8-OH-DPAT

Three-way ANOVA revealed significant effects for type of confinement factor [*F*(1,64) = 91.53, *p* < 0.05], systemic treatment factor [*F*(1,64) = 6.82, *p* < 0.05], intra-PAG treatment factor [*F*(2,64) = 12.25, *p* < 0.05], confinement × intra-PAG interaction [*F*(1,64) = 5.43, *p* < 0.05] and confinement × intra-PAG interaction × systemic treatment interactions [*F*(1,64) = 4.52, *p* < 0.05], but not for interaction type of confinement and systemic treatment [*F*(1,64) = 0.72, *p* = 0.39] and between treatments [*F*(1,64) = 1.10, *p* = 0.29].

Apart from confirming the occurrence of the OAA for almost all groups, *post hoc* analyses also revealed that chronic fluoxetine combined with intra-PAG injection of 8-OH-DPAT prevented this type of fear-induced antinociception, i.e., OA-confined animals that received fluoxetine + 8-OH-DPAT did not exhibit antinociceptive response (Figure 6B).

## Discussion

This work sought to confirm that chronic treatment with fluoxetine impairs the effects produced by the activation of 5-HT_1A_ and 5-HT_2C_ receptors located in the amygdala and PAG on OAA.

Chronic fluoxetine treatment at the dose of 20 mg/kg reduced the number of writhes in both EA- and OA-confined animals, indicating an antinociceptive effect of this SSRI (Fig. 2). At this highest dose, fluoxetine also enhanced 5-HT levels and decreased the turnover of this monoamine in the amygdala (Figures 3A,C), suggesting that this SSRI may be increasing 5-HT releasing without enhancing its synthesis. Interestingly, statistics confirmed between factor interactions for 5-HT levels, i.e. such effects on 5-HT neurotransmission occurred only in OA-confined mice, suggesting that the enhancement of the OAA produced by fluoxetine may be mediated by 5-HT action in the amygdala, in spite of the absence of statistical significance interaction between factors. Given that chronic treatment with 20 mg/kg of fluoxetine also increased 5-HT_2C_ receptor protein in the amygdala, it seems reasonable to suggest that 5-HT may be facilitating OAA through activation of 5-HT_2C_ receptors in this forebrain area. Regarding the 5-HT neurotransmission in the PAG, chronic fluoxetine did not produce any robust effect on 5-HT and 5-HIAA levels and in its turnover (Figures 3D–F).

Although no study has directly explored the effect of fluoxetine in *post mortem* tissue, *in-vivo* microdialysis has shown that acute systemic injection of the SSRI citalopram enhances 5-HT levels in the amygdala (Bosker et al., 2001; Gobert et al., 2009). A similar effect on serotonin levels was observed when the SSRI fluvoxamine was injected into the amygdala (van Der Stelt et al., 2005). Additionally, Zanoveli et al. (2010) demonstrated that chronic fluoxetine (10 mg/kg) increased 5-HT extracellular levels in the PAG of rats. Thus, it is possible that chronic fluoxetine increases serotonin levels in the amygdala of mice (present results) and also in the midbrain periaqueductal gray of rats (Zanoveli et al., 2010).

Despite chronic fluoxetine at the dose of 5.0 mg/kg did not change nociceptive response in EA- and OA-confined animals, as well as unchanged 5-HT neurotransmission either in the amygdala or in PAG, this lowest dose of fluoxetine strongly increased 5-HT_1A_ and 5-HT_2C_ receptor densities in the PAG. These apparently contrasting results on behavioral and neurochemical responses produced by 5.0 mg/kg chronic fluoxetine suggest that the changes in the 5-HT neurotransmission are dependent on the dose of this SSRI. The mechanism underlying the increases in both 5-HT_1A_ and 5-HT_2C_ receptor densities in the PAG produced by 5.0 mg/kg chronic fluoxetine remains to be determined. However, although fluoxetine is known as an SSRI (Beasley et al., 1992; Gobbi et al., 1997), subsequent findings revealed that it would also be able to block 5-HT_2C_ receptor (Bymaster et al., 2002; Carrasco and Sandner, 2005; Englander et al., 2005; Stanquini et al., 2015), and up-regulate this 5-HT_2C_ receptor subtype in the choroid plexus (Laakso et al., 1996; Palvimaki et al., 2005) and in the PAG of rats. Thus, it is likely that the prolonged blockade of 5-HT_2C_ receptors because of chronic fluoxetine treatment has up-regulated this 5-HT receptor subtype in the amygdala and PAG (Fig. 4B-D).

Corroborating the possibility of antagonistic property induced by fluoxetine at 5-HT_2__*C*_ receptors, Tavares et al. (2018) have recently demonstrated that acute systemic administration of fluoxetine or intra-amygdala injection of SB 242084 (a selective 5-HT_2C_ receptor antagonist) prevented the enhancement of OAA provoked by intra-amygdala injection of MK-212. In this context, the results of experiment 4 seem to confirm the potential role of fluoxetine as a 5-HT_2C_ antagonist receptor. Given that the activation of the 5-HT_2C_ receptors intensified OAA without changing nociceptive response in EA-confined animals, we suggest that this 5-HT receptor subtype selectively modulates pain inhibition induced by aversive situations (e.g., the OA exposure). Moreover, chronic fluoxetine prevented OAA enhancement induced by intra-amygdala MK-212. Together, these results suggest that fluoxetine could plays an antagonist role at 5-HT_2C_ receptors, impairing the facilitatory effects of MK-212 on OAA.

We do not have a clear explanation of why the lowest dose of fluoxetine (5.0 mg/kg) given chronically also increased 5-HT_1A_ receptor protein in the PAG, but not in the amygdala. However, previous studies have shown that 5-HT_1A_ and 5-HT_2C_ receptor subtypes seem to play antagonistic intracellular roles (Shih et al., 1991; Azmitia, 2007; Pytliak et al., 2011). For instance, it has been demonstrated that activation of these two serotonin receptors provokes opposite effects on behavioral and endocrine responses (Hensler and Truett, 1998; Zhang et al., 2001; Valdez et al., 2002). If so, the increased in 5-HT_2C_ receptor density following chronic fluoxetine could indirectly have increased 5-HT_1A_ receptor expression in the PAG. Such a neuroplastic mechanism would counteract an over activation of 5-HT_2C_ receptors. Although attractive this hypothesis needs further investigation.

Despite fluoxetine has not changed 5-HT1A receptor density in the amygdala, it prevented the effects of local injection of 8-OH-DPAT on OAA. Although three-way ANOVA did not reveal any statistically significant interaction effects among the factors, *post hoc* analyses pointed out that intra-amygdala injection of 8-OH-DPAT selectively attenuated OAA, i.e. it did not change nociceptive response in EA-confined animals. We expected that the blockade of 5-HT_2C_ receptors in mice chronically treated with fluoxetine would enhance (*instead of* impairing) the effects of 8-OH-DPAT in the modulation of OAA. However, fluoxetine prevented the attenuation of OAA triggered by activation of 5-HT_1A_ receptors in the amygdala. 5-HT_1A_ receptors are highly expressed in the amygdaloid complex (Hannon and Hoyer, 2008; Artigas, 2013b) and their activation has been shown to produce anxiolytic-like behavior in several animal models of anxiety (Strauss et al., 2013). If so, we suggest that the enhancement in the number of writhes in OA occurred due to the attenuation in anxiety caused by 5-HT_1A_ receptor activation.

Similarly to the results obtained in the amygdala, experiment 5 showed that intra-PAG injection of MK-212 also intensified OAA, an effect that corroborates previous studies (e.g., Baptista et al., 2012; Baptista-de-Souza et al., 2018). Again, activation of 5-HT_2C_ receptors in the PAG selectively enhanced OAA without changing nociceptive response in EA-confined animals. Once more, chronic fluoxetine prevented the effects of MK-212 on OAA, i.e., OA-confined mice that received this SSRI exhibited a similar number of writhes compared to those systemically treated with saline. Importantly, 5.0 mg/kg fluoxetine *per se* did not provoke any effect on nociception in EA-confined animals, indicating its selective effect on blocking MK-212 effects on OAA.

Intra-PAG injection of 8-OH-DPAT failed to change nociceptive response in EA- and OA-confined animals. This result corroborates a previous study showing that 5.6 or 10 nmol of 8-OH-DPAT injected into the PAG failed to alter nociception in mice exposed to the EPM (Baptista et al., 2012). Interestingly, when given with chronic fluoxetine, intra-PAG injection of 8-OH-DPAT abolished OAA. This result suggests that the blockade of 5-HT_2C_ receptor exerted by fluoxetine facilitates the anti-antinociceptive effect produced by 5-HT_1A_ receptor activation in the PAG in OA-confined mice. It is noteworthy that fluoxetine combined with intra-PAG 8-OH-DPAT did not produce any intrinsic effect on the nociception of EA-confined animals.

This assumption corroborates Valdez et al. (2002) who showed that activation of 5-HT_2_ receptor selectively attenuated 5-HT_1A_ receptor activity in neurons of the anterior cingulate cortex of rats. Therefore, it is possible to presume that blockade of 5HT_2C_ receptor produced by fluoxetine may have cooperated with 8-OH-DPAT binding at the 5HT_1A_ receptor, facilitating its effects on OAA. Together, the results of exp. 5 suggests that 5-HT modulates differentially OAA at 5-HT_1A_ and 5-HT_2C_ receptors located in the midbrain PAG. While this indolamine facilitates OAA at 5-HT_2C_ receptors, it plays an opposite effect at 5-HT_1A_ receptors on this type of fear-induced antinociception. However, present results also emphasize that the activation of the PAG 5-HT_1A_ receptors *per se* is not enough to impair OAA. To that end, it is necessary to antagonize local 5-HT_2C_ receptors to abolish it, as shown in OA-confined animals that received chronic fluoxetine treatment.

Previous studies have described that the dimerization between serotonergic receptor subtypes could explain the interplay among them. In this sense, the heterodimerization most known is 5-HT_1A_-5-HT_7_ receptors for while the heterodimers described for 5-HT_2C_ receptors involves the ER (estrogen receptor) (Herrick-Davis, 2013). Regarding these ability for dimerization of these serotonin receptors it is possible that the interaction observed in the present study could be result of the heterodimerization 5-HT_1A_-5-HT_2C_ receptors. However, this hypothesis needs to be confirmed in future molecular experiments.

Considering that the classical mechanism described with the administration of SSRI’s is to increase the extracellular levels of this neurotransmitter (Messiha, 1993) and that 5-HT_1A_ auto-receptors are directly related to the control of the serotonin release levels, resulting in the decrease of the serotonin release, these autoreceptors (5-HT_1A_) are essential to act as a compensatory mechanism, decreasing serotonin release (Deakin and Graeff, 1991). If so, we suggest that the up-regulation of 5-HT_1A_ receptor within the PAG and amygdala occurs in post synaptic neurons, since previous studies have demonstrated that chronic treatment with SSRIs provoke a 5-HT_1A_ autoreceptors desensitization and internalization (Li et al., 1996; Guptarak et al., 2010).

Considering all the above-mentioned evidence concerning the mechanisms exerted by chronic fluoxetine administration, it is conceivable to note the distinct roles of 5-HT_1A_ and 5-HT_2C_ receptors in the modulation of OAA in the amygdaloid complex and PAG. We suggest that this dissimilarity may be due to the differences in the expression of this subtype of receptors in these areas (Riad et al., 2000; Meneses et al., 2004; Popova et al., 2010). Altogether, the present findings provide evidence that fluoxetine promotes functional and quantitative changes in the 5-HT_1A_ and 5-HT_2C_ receptors located in the amygdaloid complex and PAG. If so, these neuroplasticities might be related to the necessary temporal dynamic for the therapeutic efficacy of SSRIs.

## Data Availability Statement

All datasets generated for this study are included in the article/supplementary material.

## EthicS Statement

The animal study was reviewed and approved by the Ethics Committee on the Use of Animals of the Federal University of São Carlos (Res. 046/2009; 021/2014).

## Author Contributions

DB-S, EF-C, and PC contributed to carrying out the experiments, data analysis, and drafted the manuscript. LT and LC-S contributed to the formulation and supervision of the experiments and drafted the manuscript. RN-S critically revised the manuscript. AC-S conceived the study, performed the manuscript, wrote the supervision and approved the final version to be published.

## Conflict of Interest

The authors declare that the research was conducted in the absence of any commercial or financial relationships that could be construed as a potential conflict of interest.
